# Supplementary material for: Causal interplay between lactose intolerance and gut microbiota: a combined bidirectional Mendelian randomization and in vivo validation study
Source: Front Nutr. 2026 Jun 1;13:1803337. doi: 10.3389/fnut.2026.1803337 (PMC13265576; doi:10.3389/fnut.2026.1803337)
Supplement: Supplementary file 1 [file Data_Sheet_1.zip › supplementary materials/Forward/leave one out cross validation/ebi-a-GCST90027808.finngen_R12_E4_LACTONAS.pdf]

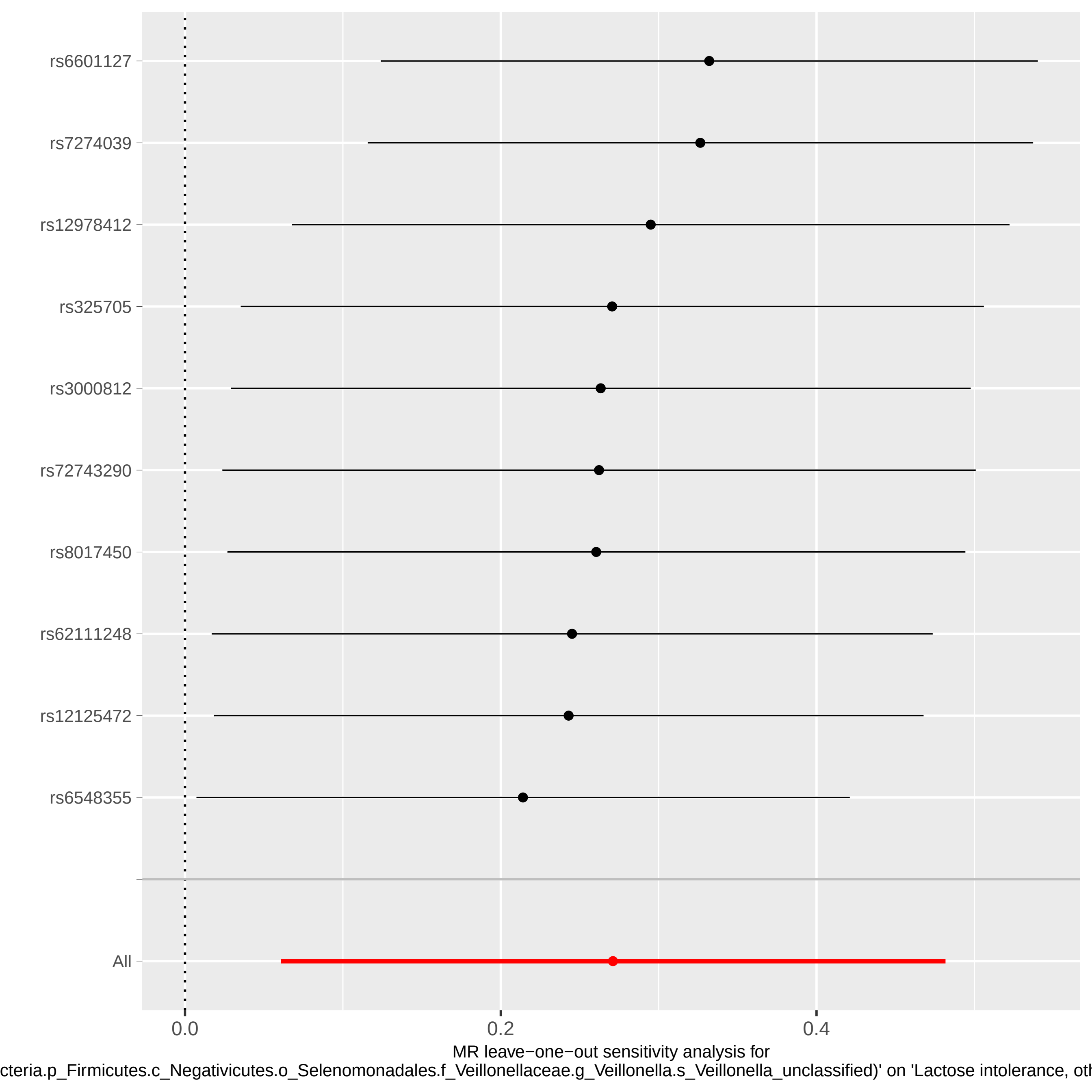
rs6601127
rs7274039
rs12978412
rs325705
rs3000812
rs72743290
rs8017450
rs62111248
rs12125472
rs6548355
All
0.0
0.2
0.4
MR leave−one−out sensitivity analysis for
cteria.p_Firmicutes.c_Negativicutes.o_Selenomonadales.f_Veillonellaceae.g_Veillonella.s_Veillonella_unclassified)' on 'Lactose intolerance, oth
